# Air speed and velocity measurements in a room with a sidewall jet

**DOI:** 10.1016/j.dib.2015.07.035

**Published:** 2015-08-06

**Authors:** M. Hurnik, M. Blaszczok, Z. Popiolek

**Affiliations:** Department of Heating, Ventilation and Dust Removal Technology, Silesian University of Technology, Konarskiego 20, 44-100 Gliwice, Poland

## Abstract

In mixing ventilation systems, diffusers are often located on side walls and supply quasi-free air jets above the occupied zone. The data presented in this paper shows a new CFD 3D benchmark with two well-defined characteristic zones in the room, i.e., the quasi-free jet zone and the occupied zone. Measurement methods adequate for air velocity and speed measurement were applied: laser Doppler anemometry for the axial velocity component in the jet and low velocity thermal anemometry for the air speed in the occupied zone. Measurements were performed in a physical scale model (1:5) of the room. The kinematic similarity criterion was fulfilled by the equality of the Reynolds numbers in the model and in the prototype. To identify boundary conditions, additional measurements were carried out in the inlet region (as close as possible to the supply opening). The CFD results validation and reporting methods applicable for the benchmark data are proposed in Hurnik et al. (2015) [1].

## Specifications table

**Table:** 

Subject area	Environmental Sciences

More specific subject area	Building, Environment, Ventilation
Type of data	Tables, figures
How data was acquired	Laser Doppler anemometer (LDA) and Pitot tube for air velocity measurement and low velocity thermal anemometer (LVTA) for air speed measurement
Data format	Raw data collected during measurement
Experimental factors	LDA and LVTA results recorded in the instruments were exported as text files and then copied to Excel spreadsheets, the results presented in this paper were recalculated from the scale model (1:5) to the prototype conditions
Experimental features	The measurements of air velocity and in the jet and speed in the occupied zone were carried out in a scale model (1:5) of the real enclosure.
Data source location	Gliwice, Poland
Data accessibility	Data are available with this paper

## Value of the data

• Turbulent diffusion of the air in the 3D jet supplied from a sidewall diffuser, impact of room partitions on the jet confinement and the air speed distribution within the occupied zone are identified.
• The benchmark data can be used for validating CFD predictions and to test different modelling aspects, such as steady or transient approach, calculation grid, turbulence model, wall treatment, boundary conditions, discretisation scheme etc.
• The data can be useful for training of CFD users and may contribute to an overall improvement of CFD prediction accuracy.

## 1 Data, experimental design, materials and methods

Data in this paper are presented in Supplementary files in the following series:

• Jet boundary conditions at the inlet, needed for identification of the inlet mean velocity, inlet volume flux, inlet momentum flux, turbulence kinetic energy and turbulence intensity based on the velocity measurement performed using a micro Pitot tube at the distance 0.094 *D*_*e*_ from the nozzle and the results of mean and standard deviation of axial and lateral velocity components measured with LDA at the distance 1.1 *D*_*e*_ from the nozzle.
• Data needed for identification of the evolution of the air mean velocity in the jet based on the mean and standard deviation of axial and lateral velocity components measured with LDA in two perpendicular planes that crossed at the middle of the supply opening.
• Data for identification of the air speed distribution in the occupied zone based on the mean and standard deviation of air speed measured with low velocity thermal anemometers.

The data reproduces a typical enclosure with dimensions corresponding to the average size office/residential room. Rectangular inlet and outlet were assumed to define boundary conditions easily. The supply nozzle construction guaranteed the uniform velocity distribution and low turbulence in the supply opening. Adequate methods for the air velocity/speed measurement in the jet and in the occupied zone were applied, i.e.: LDA for the axial velocity component in the jet and LVTA for the air speed in the occupied zone. The CFD results validation and reporting methods applicable for the benchmark data are proposed [1].

### 1.1 Tested room

**Fig. 1:**
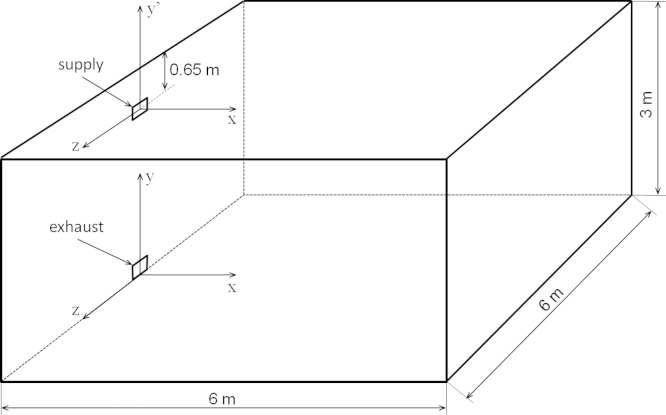
Tested ventilated room and the coordinate systems.

The measurements were carried out in isothermal conditions in a room with dimensions of 6×6×3 m^3^ (length×width×height), as shown in Fig. 1.

**Fig. 2:**
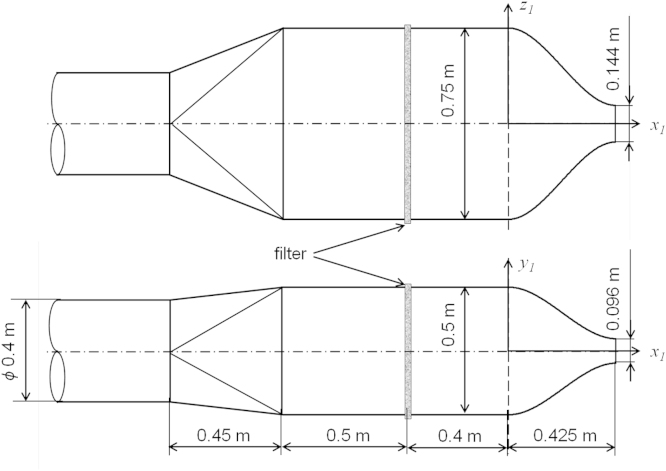
Construction of the air supply diffuser with a rectangular nozzle.

The jet was generated by a diffuser with a rectangular nozzle of the dimensions 0.144×0.096 m^2^, (equivalent diameter *D*_*e*_=0.133 m) positioned on the wall at half of the room width and at 2.35 m above the floor. The supply diffuser construction is shown in Fig. 2. The nozzle profile was calculated using the third order polynomial equation:

(1) $$\begin{array}{l} y_{1}=\pm \left[0.250-3\frac{0.250-0.048}{0.425^{2}}\cdot x_{1}^{2}+2\frac{0.250-0.048}{0.425^{3}}\cdot x_{1}^{3}\right]\\ z_{1}=\pm \left[0.375-3\frac{0.375-0.072}{0.425^{2}}\cdot x_{1}^{2}+2\frac{0.375-0.072}{0.425^{3}}\cdot x_{1}^{3}\right] \end{array}$$

The contraction ratio of the nozzle was equal 5.2.

The air supply velocity *U*_*o*_ was equal to 5.16 m/s, and the corresponding air change rate was equal to 2.4 h^−1^. The exhaust opening had the same dimensions as the supply opening and was positioned near the floor on the same wall as the supply opening. The measurements were carried out in a physical scale model (1:5) of the room. The kinematic similarity criterion was fulfilled by the equality of the Reynolds numbers in the model and the prototype, *Re*_*M*_=*Re*_*P*_=45,300. This yielded five times higher velocity and 5^2^ faster turbulent fluctuations in the scale model than in the prototype and, consequently, resulted in lower uncertainties of the air speed measurement in the occupied zone and lower uncertainties of the statistical estimators of the mean and standard deviation (uncertainties due to the limited averaging time). Although the measurements were performed in the 1:5 physical model, all of the results presented in the paper and attachments were recalculated for the prototype conditions.

### 1.2 Measurement methods

The measurements of the airflow in the presented benchmark tests were performed in two zones: the quasi-free jet zone and the occupied zone. To identify the boundary conditions, additional measurements were carried out in the inlet region (as close as possible to the supply opening). The measurement methods were selected depending on the flow characteristics in each zone.

### 1.3 Jet zone

The airflow in the jet zone is characterised by high velocity, exceeding 0.2 m/s, and relatively well-defined flow direction. In the present benchmark tests, LDA was used to measure two selected air velocity components. The LDA measurement results and the CFD prediction results could be directly compared. A water-cooled Argon Ion Laser with 4 W of power was used in the LDA system. Two velocity components were measured by a special 2D optical probe with a beam expander, which reduced the measurement volume formed by the beams׳ intersection. Different wavelengths were used to separate the measured components. Two photo-detectors with appropriate interference filters were used to detect the scattered light of the two wavelengths. The laser beam was separated into two colours: green with a wavelength equal to 514.5 nm and a power of 1.7 W and blue with a wavelength equal to 488 nm and a power of 1.3 W. The probe was equipped with a lens with a focal length of 400 mm. The dimensions of the measurement volume were 4 mm in length and 0.2 mm in diameter.

The probe was mounted on a traversing mechanism that was able to change the probe position with a resolution of ±0.01 mm. A backscattering mode was used in the velocity measurement. The light, scattered by seeding particles, was collected by the receiving lens and focused on a photo detector. Then, the signal was processed in two burst spectrum analysers (BSA). The Doppler burst was sampled (8-bit) and then processed using a true fast Fourier transform (FFT), yielding reliable and accurate measurement results even for noisy signals. Statistical estimators of the mean and standard deviation of the velocity component were calculated using a transit/residence time weighing method. The uncertainty of the velocity measurement was assessed by the manufacturer to be lower than 5% of the mean velocity value [2].

**Fig. 3:**
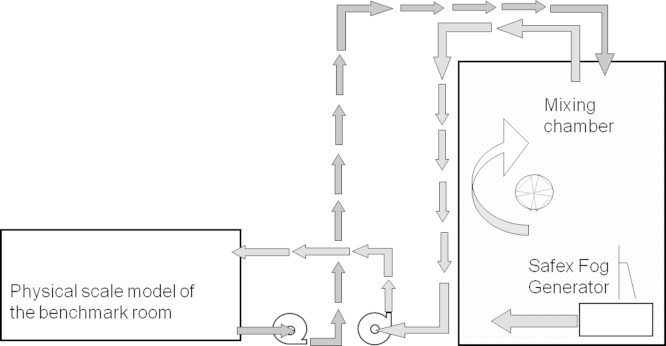
Scheme of the experimental set-up.

The benchmark room model was made of organic glass, and the air was seeded with paraffin fog. The seeding particles were mixed with the air in a special mixing chamber, as shown in Fig. 3. The particles were produced by a Safex Fog Generator located in the chamber. The supply and exhaust fans, controlled by two frequency converters, were connected to the mixing chamber and the room model. The supply velocity was controlled by a static pressure measurement at the nozzle inlet. Zero static pressure difference between the model of the room and the surrounding laboratory hall was maintained. At each measurement point, samples were collected during the period of 300 s.

Axial and lateral velocity components were measured at 222 points in two perpendicular planes that crossed at the middle of the supply opening. The velocity measurement results are presented in the file LDA&LVTA_results.xlsx.

### 1.4 Occupied zone

The air speed measurements in the occupied zone were performed in a uniform measurement grid, and 200 measurement points were located on eight horizontal planes (25 points on each plane) at heights from 0.1 m to 1.8 m and 0.5 m away from the walls. Omnidirectional hot sphere low velocity thermal anemometers (LVTA), recommended for air speed measurement in the occupied zone, were used [3]. The uncertainty of the mean speed measurement was lower than 0.01 m/s. The results of the mean speed and the speed standard deviation measurements in the occupied zone are presented in the file LDA&LVTA_results.xlsx.

### 1.5 Boundary conditions in the supply opening

The measurements were carried out in the inlet region as close as possible to the supply opening. The aim of these measurements was to identify the boundary conditions at the inlet, such as the inlet mean velocity, inlet volume flux, inlet momentum flux, turbulence kinetic energy and turbulence intensity. Two measurement series were performed. The velocity measurement was performed in a regular grid of 12.5×12.5 mm^2^ (96 points) using a micro Pitot tube at the distance *x*=12.5 mm from the nozzle (0.094 *D*_*e*_) with expanded uncertainty of 0.5%; the other measurement was carried out using LDA. The axial and lateral velocity components were measured in 418 points (a regular grid of 10×10 mm^2^) at the distance *x*=145 mm (1.1 *D*_*e*_) from the nozzle. The results are presented in the file Inlet_boundary_cond.xlsx.
